# Trends of Mental Disorders and Treatment Continuity Predictors of New Patients in the Paediatric Psychiatry Clinic of a University Hospital

**DOI:** 10.3390/ijerph18189613

**Published:** 2021-09-12

**Authors:** Ah-Rah Lee, Geon-Ho Bahn

**Affiliations:** 1 Department of Medicine, Graduate School, Kyung Hee University, Seoul 02453, Korea; preppie_i@naver.com; 2 Department of Psychiatry, College of Medicine, Kyung Hee University, Seoul 02453, Korea

**Keywords:** treatment adherence, child, adolescent, trend, outpatient clinic, new patient

## Abstract

This study analysed trends of first-time patients visiting the paediatric psychiatry clinic in a university hospital. The medical records from 2009 to 2016 of first-time patients visiting the Kyung Hee University Hospital were reviewed, focusing on children in grades 1–12. We analysed the clinical diagnosis rate of mental disorders per 100,000 in the general population by gender and grade, and the characteristics of patients who sought outpatient care more than three times. The study included 1467 participants, of which 931 were males (63.5%). The number of male patients per 100,000 population significantly decreased from 4.14 in 2009 to 2.03 in 2016. While hyperkinetic disorders had the highest prevalence in males, neurotic disorders were most frequent in females. The rate of disruptive behaviour disorders in males and mental retardation in females decreased significantly during the data collecting period. The factors affecting treatment continuity were being female, 7th–12th graders, and diagnosis of depressive, hyperkinetic, and tic disorders. Physicians should consider the new paediatric patients’ gender, grade, and expected diagnosis from their first visit to improve treatment compliance.

## 1. Introduction

There is growing interest in the mental health issues of children and adolescents [1]. Mental disorders affect a substantially greater proportion of children and adolescents in the child welfare system than in the general population. A pooled prevalence of 49% for any mental disorder was estimated through a meta-analysis [2].

In the United States, 43,283 parents of children aged 3–17 years participated in a national survey, which revealed that 7.4% of the children had current behavioural/conduct problems, 7.1% had current anxiety problems, and 3.2% had current depression [3]. The survey, which relied on parent reports, estimated that the prevalence of anxiety or depression among children aged 6 to 17 years rose from 5.4% in 2003 to 8.4% in 2011–2012 [4]. Based on a meta-analysis report summarising the prevalence of common mental disorders among adolescents aged between 10 and 19 years, the global prevalence of common mental disorders measured using the 12-item General Health Questionnaire, with cut-off points of 4 and 3, was 25% and 31%, respectively [5]. A child and adolescent mental health survey in Seoul reported the estimated prevalence of full-syndrome and subthreshold Diagnostic and Statistical Manual of Mental Disorders, Fourth Edition (DSM-IV) disorders as 16.2% and 28.1%, respectively [6].

Interestingly, the prevalence rate of mental disorders estimated through epidemiological investigations and the prevalence rate of diagnosis observed in clinical practice may be different. The diagnosis prevalence rate of mental disorders was 1.95–2.38% based on the Korean Health Insurance Review and Assessment Service (HIRA) sample data for children aged 0–18 years during 2010–2015 [7]. These gaps can also be identified in individual disorders. While attention deficit/hyperactivity disorder (ADHD) is reported among 5–8% of children worldwide, the average annual diagnosis prevalence of ADHD in Korean children is 0.357%. Similarly, though the prevalence of disruptive mood dysregulation disorder is estimated as 2–5% in DSM-5 [8], the weighted prevalence was 0.3–0.76% in Taiwanese children, as observed in a national epidemiological study [9]. These differences can also be explained by variables such as comorbid mental disorders or the preference for psychotropic medication [10].

Interest in mental disorders among children and adolescents has heightened; however, there is not enough data based on recent clinical practice. Several studies have reported limited data for specific years and conditions on clinical aspects of first-time paediatric patients, but few have identified changes and long-term trends for the entire population.

The present study aimed to bridge this gap in existing research by analysing the trends of clinical diagnoses in a university hospital’s paediatric psychiatry outpatient clinic compared to the total population and the long-term follow-up of new patients for eight years.

## 2. Materials and Methods

### 2.1. Participants

This study included patients from grades 1–12 who were first-time patients at the outpatient clinic of paediatric psychiatry, Kyung Hee University Hospital, Seoul, Korea during an eight-year period from January 2009 to December 2016. Kyung Hee University Hospital is a general hospital with about 800 beds and is one of more than 30 university hospitals in the Seoul metropolitan area. Three paediatric psychiatrists and a clinical psychologist are in charge of outpatient care for youth. Infants and pre-schoolers between the ages of 0–6 years were excluded in this study because they needed to be monitored for a certain period for an accurate diagnosis and were thus difficult to diagnose definitely [7]. Data collecting was set to 2009–2016 because the hospital’s electronic medical records system was changed before and after the data collection period. However, there were no changes in the psychiatrists and clinical psychologists during that period, thereby ensuring consistency between the medical record system and the medical staff.

### 2.2. Methods

During the study period, gender, grade, and diagnosis were retrospectively analysed using medical records of first-time patients by year. To analyse the factors influencing treatment continuity, participants with fewer than three outpatient visits were classified as the early dropout group, while those with three or more outpatient visits were considered the treatment continuity group; this was based on the study on early dropout factors by Pelkonen et al. [11]. Patients were diagnosed by paediatric psychiatrists and a clinical psychologist by the Korean Standard Classification of Diseases (KCD) [12], which is based on the International Classification of Diseases [13].

The age groups were analysed using three-year intervals (grades 1–3, 4–6, 7–9, 10–12) based on a previous study reporting diagnosis prevalence of mental disorders in the 0–18 years age group [7].

To compensate for the limitations of single hospital data, the diagnosis rate for the total population was calculated by reflecting the total population in the middle of the year [14]. In other words, the clinical diagnosis of mental disorders was analysed based on the total population aged 6–18 years per year.

The KCD codes for diagnosis were limited to mental disorders (F codes), symptoms and signs and abnormal clinical and laboratory findings, not elsewhere classified (R codes), and factors influencing health status and contact with health services (Z codes) (Table S1). Psychiatrists are often unable to make a clinical diagnosis during a child’s first visit to a psychiatric clinic. Therefore, a tentative diagnosis was attached with an R or Z code based on the chief complaints. If the clinical diagnosis was determined during the treatment, an F code was attached. Once an F code diagnosis was made, the R and Z codes were not included in the analysis. In cases where the treatment was discontinued before the F code diagnosis was made, either the R or Z code diagnosis was included in the analysis.

In the case of mental disorders, six higher diagnostic groups were established in the order of frequency based on HIRA sample data research [9] and the preliminary analysis of this data (Table S2). Hyperkinetic disorders (F90) and tic disorders (F95) belonged to a single diagnostic group. The other four groups of diagnoses were classified together with similar disorders: neurotic disorders (F40–48), depressive disorders (F32, 33), disruptive behaviour disorders (F91–94), and mental retardation (F70–79). Disorders that affect a small number of patients were classified as ‘others’ which include the following diagnoses: psychotic disorders (F20–29), bipolar disorders (F30, 31), eating and sleep disorders (F50, 51), personality disorders (F60–65), learning and developmental disorders (F80–84), and unspecified mental disorder (F99). The analysis of diagnosis per person allowed for redundancy.

### 2.3. Statistical Analysis

The changes in the diagnosis prevalence of mental disorders from 2009 to 2016 by gender, grade, and year were examined according to the rate of increase or decrease in the annual average, and the significance of the trends was estimated using a trend test. The prevalence rate was converted to a percentage of the F code diagnosed by psychiatrists per 100,000 population included in each group as designated by the total mid-year population.

To investigate the factors affecting treatment continuity, the patients were divided into groups based on the number of outpatient visits, and the most effective logistic regression model was selected through the stepwise method based on gender, grade, estimated diagnosis, and the number of diagnosis redundancies. A generalised linear model with the interaction term of year and grade was estimated to compare the annual growth rate of each grade by gender.

The statistical significance was set at 0.05 for all analyses, and the data were analysed using R 4.0.2. in SAS 9.4 (Statistical Analysis System version 9.4, SAS Institute, Cary, NC, USA). 

## 3. Results

Of the 1467 participants, 931 (63.5%) were male and 536 (36.5%) were female. Between 2009 and 2016, the total number of cases per 100,000 population of the age group (6–18 years) decreased significantly from 3.24 to 1.88, and the number of male patients decreased significantly from 4.14 to 2.03 (Table 1). The number of female patients decreased from 2.24 to 1.72, which was not statistically significant. The overall ratio of male and female patients decreased from 1.87 to 1.29 during the study period, which was not statistically significant. In the overall distribution of participants by grade, the largest proportion of patients were in grades 1–3. In terms of year-over-year changes, changes in grades 1–3 were higher than those for other grades in 2009, 2011, and 2013, and changes in grades 10–12 were higher than those for others in 2010, 2012, and 2014–2016. The number of males decreased significantly in grades 1–3, 7–9, and 10–12. The number of females showed a significant decline in grades 4–6 and 7–9. Using a generalised linear model with year and grade as the interaction terms revealed that the number of female participants in grades 10–12 showed a significant increase compared to those in grades 1–3.

**Figure 1:**
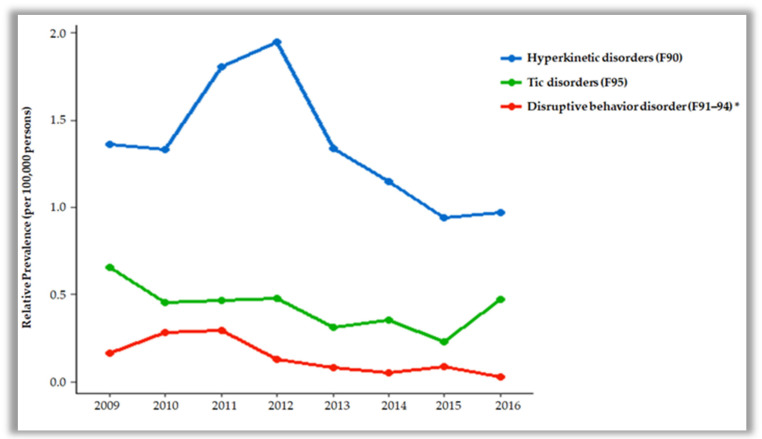
Distribution of mental disorders in male patients by year (*p* value of slope, * <0.05).

**Figure 2:**
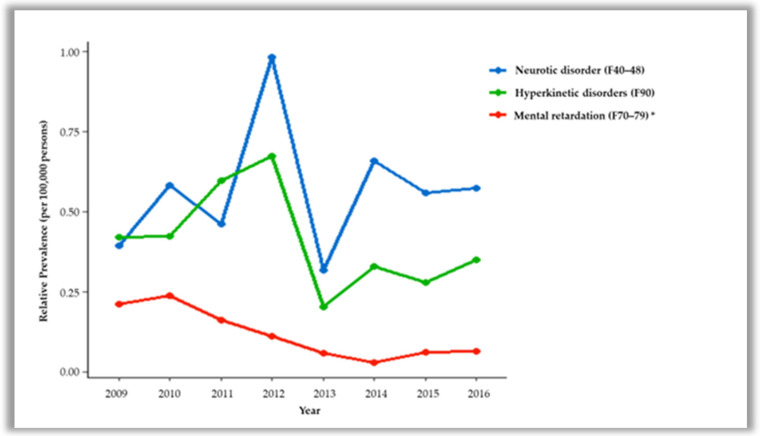
Distribution of mental disorders in female patients by year (*p* value of slope, * <0.05).

In terms of the distribution of the diagnostic codes in male participants, hyperkinetic disorders (F90) were the highest, followed by neurotic disorders (F40–48), tic disorders (F95), psychotic disorders (F20–29), bipolar disorders (F30, 31), eating and sleep disorders (F50, 51), personality disorders (F60–69), specific developmental disorders of speech and language (F80), specific developmental disorders of scholastic skills (F81), pervasive developmental disorders (F84), unspecified mental disorder (F99), mental retardation (F70–79), depressive disorders (F32, 33), and disruptive behaviour disorders (F91–94). F90 had the highest diagnostic rate during the data-collecting period, but was on the decline. F91–94 diagnoses significantly decreased with each year (Figure 1). In females, the diagnostic distribution was followed by F40–48, F90, F32, F33, others, F70–79, F95, and F91–94. The most common single diagnosis in females was other anxiety disorders (F41), followed by depressive episodes (F32), somatoform disorders (F45), and F90. F70–79 diagnoses significantly decreased with the year (Figure 2). 

The total numbers of R and Z codes were 317 (males 62.8%) and 98 (males 68.4%), respectively.

Of the 1467 participants, 645 (45.0%) (423 males and 222 females) dropped out early from the outpatient clinic (Table S3). Factors predicting continuity of follow-up were patients who were female, in grades 7–12, with mental disorders, including depressive disorder (F32, 33), hyperkinetic disorders (F90), and tic disorders (F95), and with one or more clinical diagnosis (Table 2).

## 4. Discussion

In this study, a significant decline in the total number of first-time and male patients was observed, even if the statistics are corrected to reflect the decline in the birth rate in the Republic of Korea. These results are consistent with the decreased prevalence of diagnosis of mental disorders in children and adolescents under 19 years of age reflected in HIRA statistics [7]. These trends are contrary to the increase in the prevalence of mental disorders in children and adolescents in screening-oriented epidemiological studies [15]. This suggests that there may be differences in estimated prevalence and diagnosis rate in clinical practice, depending on epidemiological investigation methods or national healthcare systems. Two factors could have contributed to this trend: first, the Adolescent Personality and Mental Health Problems Screening Questionnaire, conducted annually for the 1st, 4th, 7th, and 10th graders across the whole country in the Republic of Korea since 2012 and the increased number of treatment-linked cases; second, parents’ awareness of youth mental disorders through media or public education [1].

Gender ratio of mental disorders changes during the transition from childhood to adulthood because of biological and environmental factors. Based on 2011 HIRA data, in a study of the population under the age of 19, the ratio of male to female patients with mental disorders was 1.68:1 [16]. This study also showed that the percentage of male patients was higher than that of female patients. The sample data from the HIRA of 1,375,842 people in 2011 revealed that the male-to-female ratio for those aged 19–30 years was 48.1 to 51.9 [17]. A cross-national meta-analysis of mental disorders in the World Health Organisation World Mental Health Surveys also revealed that the prevalence of mental disorders is 1.1 times higher in women than in men [18]. What is noteworthy here is the cause of this reversal of the gender ratio. One of the reasons for this significant narrowing of gender differences was related to depressive disorders and the variation of gender role traditionalism [18].

In this study, the number of male patients from grades 1 to 12 and female patients from grades 1 to 9 decreased. Interestingly, the increase in the number of female patients in grades 10–12 was statistically significant compared to other grades when the number of patients in grades 1–3 was a reference, though the increase in the number of female patients in grades 10–12 over the year was not statistically significant. One of the contributing factors for the trend observed in this study was the different distribution of mental disorders in female patients compared to males. The most common diagnosis in female patients in this study was F41, followed by F32, F45, and F90. The lifetime diagnosis of anxiety or depression among children aged 6 to 17 years has increased based on the US parent report survey [4]. While current anxiety increased significantly, current depression did not change. In a study on the prevalence of paediatric mental disorders in Taiwan, the lifetime prevalence was the highest for anxiety disorder, followed by ADHD, sleep disorder, and tic disorders [19]. Concurrent comorbidity and homotypic and heterotypic continuity from age 9 through 16 years were more marked in girls than in boys, and girls had a higher incidence of depressive disorders as they grew [20]. In Kim’s study [21], the number of patients from grades 1–3 was more than that from any other grades from 2004 to 2009, and that from grades 10–12 was more than that from any other grades from 2010 to 2013. During that period, the incidence of depressive disorders increased among grades 7 to 12.

The most common diagnosis in male patients in this study was F90. One of the reasons why the grades 1–3 group is larger than other grades in the psychiatric outpatient clinic may be because the group has a higher diagnosis and treatment rate of ADHD patients [7]. From three population-based cohorts and a meta-analysis, it is evident that children and adolescents who are relatively younger than their classmates have a higher risk of receiving an ADHD diagnosis [22]. According to the Center for Disease Control and Prevention in the United States, ADHD was the most frequent mental disorder among children under the age of 18 [23]. ADHD was the most common mental disorder in the population under the age of 19 based on the HIRA sample data, followed by other anxiety disorders (F41), depressive episodes (F32), somatoform disorders (F45), reaction to severe stress, and adjustment disorders (F43) [16]. A long-term comparison over a 26-year period (1980–2005) from a tertiary care centre for child and adolescent psychiatric service in India revealed an increase in registration with affective illnesses in ages 10–15, which is reflective of a worldwide trend towards an earlier onset and increased prevalence of affective illnesses [24]. In a study of the prevalence rate of overall mental disorders among adolescents across three consecutive years in Taiwan, the rates for ADHD and phobia decreased and those for major depression and substance use disorders increased [25]. Conduct disorder and ADHD were more prevalent among boys, while major depression and phobia were higher among girls.

A significant decrease in F91–94 was observed among male patients over the years, while F70–79 showed a decreasing trend in female patients in this study. A comparison of the clinical profiles of patients in child psychiatry in Bahrain between 1981–1982 and 2011–2012 showed that the prevalence rate of conduct disorders and anxiety disorders was lower [26]. The reason for this drop in prevalence is more likely to be due to other reasons than due to a real decrease, such as in those diagnosed with ADHD or learning disorders instead of conduct disorders. In this study, if conduct disorder and ADHD coexist in the same patient, they may have been diagnosed with ADHD. The prevalence of conduct disorders also declined between 1998 and 2013–2014 among younger males according to a study in Australia, primarily due to a decline in the prevalence among males living in two-parent families [27]. A significant reduction in F70–79 may be due to early detection and early intervention among individuals with mental retardation during their preschool period compared to the past, resulting in improved adaptability [16]. However, the reason for the significant decline in females alone in this study should be identified in future studies.

In this study, significant factors for treatment continuity were females, higher grades, one or more clinical diagnosis, and disorders such as depressive disorders, hyperkinetic disorders, and tic disorders. One of the causes that affect the duration of treatment among those in higher grades and in females may be associated with increased anxiety and depressive disorders in the concerned gender and age group [9]. A study based on inpatient adolescents found that being female was a predictor of change in global functioning [28]. Pelkonen et al. [11] also reported that not having a mood disorder and not using psychotropic medication were associated with early dropout of adolescents from outpatient psychiatric treatment. The younger the patients, the more likely they were to drop out early without commencement of pharmacotherapy in ADHD [29]. Younger children are at higher risk of receiving suboptimal care with psychotropic medications [30], and their medication adherence may reduce depending on their parents’ adherence [31]. These results suggest that the factors of treatment compliance may vary depending on the target disorders, culture, age, and gender of the subjects, or the research design.

This study has several limitations. First, as a descriptive study, the results and conclusions are limited to this sample. The participants of this study are outpatients at one university hospital, and one should be careful while applying the findings of this study to the entire general population. However, since it is based on medical records of the same medical staff in a single institution, consistency in care can be an advantage.

Second, this study is a retrospective review of medical records and does not apply the planned diagnostic tools as in prospective studies. Therefore, it has the limitation of application and the difficulty of identifying the causes of the results. In future, it may be better to conduct a prospective study to estimate the prevalence. Although this study adopted a retrospective perspective, the fact that the same paediatric psychiatrists and the same clinical psychologist conducted diagnostic procedures also provides the advantage of increasing accuracy and consistency in diagnosis.

Third, the results may reflect certain bias. It is possible that the patients referred to the psychiatry clinic of a university hospital have more serious symptoms or different associated characteristics than those not requiring the university hospital clinic. Moreover, such patients may have been more likely to have pre-existing psychiatric disorders because of referral bias.

Despite these limitations, this is the first study in Korea wherein the trends in outpatients have been verified by reflecting changes in the overall population. The identification of treatment continuity predictors in first-time patients is also a strength of this study. The study is meaningful in that patients with treatment continuity were followed-up with for about eight years at the same outpatient clinic. 

Follow-up studies involving other university hospitals and private clinics are needed to overcome these shortcomings and to apply the results of this study to extrapolate mental health problems in the general child and adolescent populations.

## 5. Conclusions

While the number of first-time patients in a university hospital’s paediatric psychiatry department significantly declined over eight years, the number of older female patients did not decrease. Furthermore, while hyperkinetic disorders were commonly found in male patients, anxiety and depressive disorders were commonly observed in females. The analysis of treatment continuity factors suggests that being female, in a higher grade, and having several mental disorders are important predictors. While the present study adopted a retroactive perspective, analysing trends of clinical diagnoses in a university hospital’s paediatric psychiatry outpatient clinic compared to the total population may prove beneficial in predicting future outpatient trends.

## Figures and Tables

**Table 1:** Number of first-time patients according to year, gender, and grades (per 100,000).

Sex	Grades	2009	2010	2011	2012	2013	2014	2015	2016	Estimate	CI		*p*-Value
Total	1–3	4.03	4.04	4.01	3.19	3.42	2.17	2.70	2.15	−0.30	−0.43	−0.17	0.0015
	4–6	2.31	2.80	2.25	2.80	1.31	1.36	0.93	1.54	−0.23	−0.41	−0.04	0.0262
	7–9	3.74	2.93	2.16	2.61	2.19	1.83	1.24	1.60	−0.30	−0.43	−0.16	0.0017
	10–12	2.92	3.47	1.30	2.63	1.70	2.07	2.08	2.20	−0.13	−0.38	0.13	0.2649
	Subtotal	3.24	3.29	2.33	2.78	2.08	1.86	1.72	1.88	−0.23	−0.34	−0.12	0.0022
Male	1–3	5.66	5.45	5.09	4.60	5.78	3.49	3.95	2.64	−0.38	−0.64	−0.13	0.0106
	4–6	3.00	3.80	3.20	3.61	1.64	1.54	1.02	2.42	−0.30	−0.60	0.00	0.0525
	7–9	4.47	2.84	2.45	2.63	2.73	2.00	1.24	1.97	−0.32	−0.53	−0.11	0.0106
	10–12	3.54	3.90	1.40	2.73	1.95	1.82	1.90	1.38	−0.29	−0.55	−0.03	0.0324
	Subtotal	4.14	3.93	2.88	3.29	2.84	2.13	1.93	2.03	−0.33	−0.44	−0.21	0.0004
Female	1–3	2.25	2.50	2.84	1.66	0.87	0.75	1.36	1.63	−0.20	−0.43	0.02	0.0713
	4–6	1.55	1.71	1.20	1.92	0.95	1.16	0.83	0.58	−0.15	−0.26	−0.03	0.0220
	7–9	2.90	3.02	1.83	2.59	1.60	1.64	1.24	1.19	−0.27	−0.40	−0.13	0.0028
	10–12	2.22	2.99	1.19	2.52	1.41	2.34	2.29	3.10	0.06	−0.21	0.33	0.6088
	Subtotal	2.24	2.57	1.71	2.21	1.24	1.56	1.49	1.72	−0.12	−0.26	0.01	0.0650
Male/Female ratio		1.87	1.56	1.72	1.56	2.50	1.50	1.42	1.29	−0.05	−0.20	0.09	0.4005

**Table 2:** Factor analysis for the treatment continuity defined as more than three outpatient visits.

Item			OR	CI (95%)		*p*-Value *
				Lower	Upper	
Sex		Male	1.00			
		Female	1.36	1.07	1.74	0.0125
Grades		1–3	1.00			
		4–6	1.04	0.75	1.44	0.8261
		7–9	1.39	1.01	1.90	0.0422
		10–12	1.81	1.30	2.52	0.0005
Diagnosis	Depressive episode (F32, 33)		1.65	1.08	2.51	0.0198
	Hyperkinetic disorders (F90)		3.14	2.32	4.26	<0.0001
	Tic disorders (F95)		2.83	1.89	4.24	<0.0001
Number of clinical diagnoses #		0 1	1.00 2.02	1.44	2.82	<0.0001
		≥2	1.63	1.02	2.61	0.0416

* Multiple logistic regression tests. **#** Number of clinical diagnoses was focused on F32, F33, F40–48, F70–79, F90, F91–94, and F95.

## Data Availability

Not applicable.

## Supplementary Materials

The following are available online at https://www.mdpi.com/article/10.3390/ijerph18189613/s1. Table S1. Diagnoses with F, R, and Z codes; Table S2. Number of first-time patients with clinical diagnosis by year; Table S3. Clinical characteristics of early dropout group and treatment continuity group by gender and grade (%).

## References

[1] Song J., Kweon Y.S., Hong S.H., Kim J., Chun K.H., Bahn G.H., Yook K., Shin D.W., Hong H.J. (2020). Characteristics of first visit pediatric patients with suicidal ideation and behavior: An 8-year retrospective chart review. J. Korean Acad. Child Adolesc. Psychiatry.

[2] Bronsard G., Alessandrini M., Fond G., Loundou A., Auquier P., Tordjman S., Boyer L. (2016). The prevalence of mental disorders among children and adolescents in the child welfare system: A systematic review and meta-analysis. J. Med..

[3] Ghandour R.M., Sherman L.J., Vladutiu C.J., Ali M.M., Lynch S.E., Bitsko R.H., Blumberg S.J. (2019). Prevalence and treatment of depression, anxiety, and conduct problems in U.S. children. J. Pediatr..

[4] Bitsko R.H., Holbrook J.R., Ghandour R.M., Blumberg S.J., Visser S.N., Perou R., Walkup J.T. (2018). Epidemiology and impact of healthcare provider diagnosed anxiety and depression among US children. J. Dev. Behav. Pediatr..

[5] Silva S.A., Silva S.U., Ronca D.B., Gonçalves V.S.S., Dutra E.S., Carvalho K.M.B. (2020). Common mental disorders prevalence in adolescents: A systematic review and meta-analyses. PLoS ONE.

[6] Park S., Kim B.N., Cho S.C., Kim J.W., Shin M.S., Yoo H.J. (2015). Prevalence, correlates, and comorbidities of DSM-IV psychiatric disorders in children in Seoul, Korea. Asia Pac. J. Public Health.

[7] Lee S.Y., Bahn G.H. (2020). Patterns of the diagnosis prevalence of psychiatric disorders in the population aged 0–18 years based on the nationwide insurance sample data. J. Korean Acad. Child Adolesc. Psychiatry.

[8] American Psychiatric Association (2013). Diagnostic and Statistical Manual of Mental Disorders.

[9] Lin Y.J., Tseng W.L., Gau S.S. (2021). Psychiatric comorbidity and social adjustment difficulties in children with disruptive mood dysregulation disorder: A national epidemiological study. J. Affect. Disord..

[10] Findling R.L., Zhou X., George P., Chappell P.B. (2021). Diagnostic trends and prescription patterns in disruptive mood dysregulation disorder and bipolar disorder. J. Am. Acad. Child Adolesc. Psychiatry.

[11] Pelkonen M., Marttunen M., Laippala P., Lönnqvist J. (2000). Factors associated with early dropout from adolescent psychiatric outpatient treatment. J. Am. Acad. Child Adolesc. Psychiatry.

[12] KOICD Korean Standard Classification of Diseases, KCD-7, 2016. https://www.koicd.kr/kcd/guide.do.

[13] World Health Organization (1992). The ICD-10 Classification of Mental and Behavioural Disorders: Clinical Descriptions and Diagnostic Guidelines.

[14] Statistics Korea Population in the Middle of the Year. https://kosis.kr/statHtml/statHtml.do?orgId=101&tblId=DT_1B040M1&conn_path=I2.

[15] Polanczyk G.V., Salum G.A., Sugaya L.S., Caye A., Rohde L.A. (2015). Annual Research Review: A meta-analysis of the worldwide prevalence of mental disorders in children and adolescents. J. Child Psychol. Psychiatry.

[16] Hwangbo R., Chang H., Hong M., Cho S., Bahn G.H. (2016). The diagnostic distribution of psychiatric disorders among the population under 19 years Old: Based on the national insurance data. J. Korean Acad. Child Adolesc. Psychiatry.

[17] Hwangbo R., Chang H., Bahn G.H. (2020). Diagnostic distribution of psychiatric disorders among Korean young adults. J. Korean Acad. Child Adolesc. Psychiatry.

[18] Seedat S., Scott K.M., Angermeyer M.C., Berglund P., Bromet E.J., Brugha T.S., Demyttenaere K., de Girolamo G., Haro J.M., Jin R. (2009). Cross-national associations between gender and mental disorders in the World Health Organisation World Mental Health Surveys. Arch. Gen. Psychiatry.

[19] Chen Y.L., Chen W.J., Lin K.C., Shen L.J., Gau S.S. (2019). Prevalence of DSM-5 mental disorders in a nationally representative sample of children in Taiwan: Methodology and main findings. Epidemiol. Psychiatr. Sci..

[20] Costello E.J., Mustillo S., Erkanli A., Keeler G., Angold A. (2003). Prevalence and development of psychiatric disorder in childhood and adolescence. Arch. Gen. Psychiatry.

[21] Kim H., Jung S., Jung C. (2015). The distributional changes in the first-visit psychiatric child and adolescent outpatients at a university hospital over a ten-year period. J. Korean Acad. Child Adolesc. Psychiatry.

[22] Caye A., Petresco S., de Barros A.J.D., Bressan R.A., Gagelha A., Gonçalves H., Manfro A.G., Matijasevich A., Menezes A.M.B., Miguel E.C. (2020). Relative age and attention-deficit/hyperactivity disorder: Data from three epidemiological cohorts and a meta-analysis. J. Am. Acad. Child Adolesc. Psychiatry.

[23] Perou R., Bitsko R.H., Blumberg S.J., Pastor P., Ghandour R.M., Gfroerer J.C., Hedden S.L., Crosby A.E., Visser S.N., Schieve L.A. (2013). Centers for Disease Control and Prevention (CDC) Mental health surveillance among children-United States, 2005–2011. MMWR.

[24] Malhotra S., Biswas P., Sharan P., Grover S. (2007). Characteristics of patients visiting the child & adolescent psychiatric clinic: A 26-year study from north India. J. Indian Assoc. Child Adolesc. Ment. Health.

[25] Gau S.S., Chong M.Y., Chen T.H.H., Cheng A.T.A. (2005). A 3-year panel study of mental disorders among adolescents in Taiwan. Am. J. Psychiatry.

[26] Al-Ansari A.M. (2015). Characteristics of child and adolescent populations visiting a public child and adolescent psychiatric clinic in Bahrain: A 30-year comparative analysis. Arab. J. Psychiatry.

[27] Sawyer M.G., Reece C.E., Sawyer A.C.P., Johnson S.E., Lawrence D. (2018). Has the prevalence of child and adolescent mental disorders in Australia changed between 1998 and 2013 to 2014?. J. Am. Acad. Child Adolesc. Psychiatry.

[28] Kennedy J., Hembry P., Green D., Skuse D., Lewis S. (2020). Predictors of change in global psychiatric functioning at an inpatient adolescent psychiatric unit: A decade of experience. Clin. Child Psychol. Psychiatry.

[29] Hong M., Lee W.H., Moon D.S., Lee S.M., Chung U., Bahn G.H. (2014). A 36 month naturalistic retrospective study of clinic-treated youth with attention-deficit/hyperactivity disorder. J. Child Adolesc. Psychopharmacol..

[30] Dinnissen M., Dietrich A., van der Molen J.H., Verhallen A.M., Buiteveld Y., Jongejan S., Troost P.W., Buitelaar J.K., Hoekstra P.J., van den Hoofdakker B.J. (2020). Prescribing antipsychotics in child and adolescent psychiatry: Guideline adherence. Eur. Child Adolesc. Psychiatry.

[31] Nagae M., Nakane H., Honda S., Ozawa H., Hanada H. (2015). Factors affecting medication adherence in children receiving outpatient pharmacotherapy and parental adherence. J. Child Adolesc. Psychiatr. Nurs..

