# Supplementary material for: Trends of Mental Disorders and Treatment Continuity Predictors of New Patients in the Paediatric Psychiatry Clinic of a University Hospital
Source: Int J Environ Res Public Health. 2021 Sep 12;18(18):9613. doi: 10.3390/ijerph18189613 (PMC8464688; doi:10.3390/ijerph18189613)
Supplement: Supplementary file 1 [file ijerph-18-09613-s001.zip › ijerph-1329582-supplementary.pdf]

Table S1. Diagnoses with F, R, and Z codes.

Groups		KCD-7 codes	Diagnosis
Depressive symptom		F32	Depressive episode
		F33	Recurrent depressive disorder
Neurotic symptom		F40	Phobic anxiety disorders
		F41	Other anxiety disorders
		F42	Obsessive-compulsive disorder
		F43	Reaction to severe stress, and adjustment disorders
		F44	Dissociative [conversion] disorders
		F45	Somatoform disorders
		F48	Other neurotic disorders
Low intelligence		F70–79	Mental retardation
Hyperkinetic problems		F90	Hyperkinetic disorders
Disruptive behaviors		F91	Conduct disorders
		F92	Mixed disorders of conduct and emotions
		F93	Emotional disorders with onset specific to childhood
		F94	Disorders of social functioning with onset specific to childhood and adolescence
Tic symptoms		F95	Tic disorders
Others	Psychotic symptoms	F20–29	Schizophrenia, schizotypal and delusional disorders
	Mood-bipolarity	F30	Manic episode
		F31	Bipolar affective disorder
	Eating and sleep problems	F50	Eating disorders
		F51	Nonorganic sleep disorders
	Personality problem	F60–69	Personality disorders
	Learning and developmental problems	F80	Specific developmental disorders of speech and language
		F81	Specific developmental disorders of scholastic skills
		F84	Pervasive developmental disorders
	Unspecified mental disorder	F99	
Symptoms, signs and abnormal clinical and laboratory findings, NEC		R40–R46	Symptoms and signs involving cognition, perception, emotional state and behaviour
		R47–R49	Symptoms and signs involving speech and voice
		R50–R69	General symptoms and signs
		R90–R94	Abnormal findings on diagnostic imaging and in function studies, without diagnosis
Factors influencing health status and contact with health services		Z30–Z39	Persons encountering health services in circumstances related to reproduction
		Z40–Z54	Persons encountering health services for specific procedures and health care
		Z55–Z65	Persons with potential health hazards related to socioeconomic and psychosocial circumstances
		Z70–Z76	Persons encountering health services in other circumstances

KCD-7: Korean Standard Classification of Diseases-7.

Table S2. Number of first-time patients with clinical diagnosis by year.

Diagnosis	2009	2010	2011	2012	2013	2014	2015	2016	Estimate	CI		*p*-Value
F32–33	15	24	14	23	15	18	11	28	0.014	−0.020	0.047	0.3655
F40–48	31	45	37	65	22	28	37	28	−0.007	−0.077	0.063	0.8214
F70–79	23	21	19	11	10	3	7	10	−0.028	−0.048	−0.007	0.0155
F90	74	72	96	101	58	53	42	44	−0.061	−0.147	0.025	0.1355
F91–94	11	19	16	8	7	5	4	4	−0.022	−0.039	−0.005	0.0187
F95	31	24	27	27	16	13	11	20	−0.022	−0.048	0.004	0.0805
Other	25	25	17	18	13	10	15	12	−0.019	−0.035	−0.004	0.0220

F32, 33: Depressive disorder; F40–-48: Neurotic disorder; F70–79: Mental retardation; F90: Hyperkinetic disorders; F91–94: Disruptive behaviour disorder; F95: Tic disorders; Other; F20–29, F30, F31, F50, F51, F60–69, F80, F81, F84, F99, F20–29: Psychotic disorder; F30, 31: Bipolar disorder; F50, F51: Eating and Sleep disorder; F60–69: Personality disorder; F80–84: Learning and developmental disorder; F99: Unspecified mental disorder.

Table S3. Clinical characteristics of early dropout group and treatment continuity group by gender and grade (%).

		Total (*n* = 1432)		Male (*n* = 911)		Female (*n* = 521)		*p*-Value *	*p*-Value *
Early dropout group		645	(45.0)	423	(46.4)	222	(42.6)	<0.0017	
Grade	1–3	191	(29.6)	143	(33.8)	48	(21.6)		
	4–6	136	(21.1)	94	(22.2)	42	(18.9)		
	7–9	167	(25.9)	98	(23.2)	69	(31.1)		
	10–12	151	(23.4)	88	(20.8)	63	(28.4)		
Treatment continuity group		787	(55.0)	488	(53.6)	299	(57.4)	<.0001	<0.0001
Grade	1–3	214	(27.2)	156	(32.0)	58	(19.4)		
	4–6	142	(18.0)	98	(20.1)	44	(14.7)		
	7–9	202	(25.7)	118	(24.2)	84	(28.1)		
	10–12	229	(29.1)	116	(23.8)	113	(37.8)		

* chi-square test, ** CochranMantel-Haenszel test.
